# Inhibition of cytokine response to TLR stimulation and alleviation of collagen‐induced arthritis in mice by *Schistosoma japonicum* peptide SJMHE1

**DOI:** 10.1111/jcmm.12991

**Published:** 2016-09-28

**Authors:** Xuefeng Wang, Li Li, Jun Wang, Liyang Dong, Yang Shu, Yong Liang, Liang Shi, Chengcheng Xu, Yuepeng Zhou, Yi Wang, Deyu Chen, Chaoming Mao

**Affiliations:** ^1^ Department of Central LaboratoryThe Affiliated Hospital of Jiangsu UniversityZhenjiangChina; ^2^ Department of Nuclear Medicine and Institute of OncologyThe Affiliated Hospital of Jiangsu UniversityZhenjiangChina; ^3^ Department of Nuclear MedicineThe Affiliated People's Hospital of Jiangsu UniversityZhenjiangJiangsuChina; ^4^ Clinical LaboratoryHuai'an Hospital Affiliated of Xuzhou Medical CollegeHuaianJiangsuChina

**Keywords:** *Schistosoma japonicum* peptide SJMHE1, cytokine response, inhibition, toll‐like receptor, collagen‐induced arthritis, alleviation

## Abstract

Helminth‐derived products have recently been shown to prevent the development of inflammatory diseases in mouse models. However, most identified immunomodulators from helminthes are mixtures or macromolecules with potentially immunogenic side effects. We previously identified an immunomodulatory peptide called SJMHE1 from the HSP60 protein of *Schistosoma japonicum*. In this study, we assessed the ability of SJMHE1 to affect murine splenocytes and human peripheral blood mononuclear cells (PBMCs) stimulated by toll‐like receptor (TLR) ligands *in vitro* and its treatment effect on mice with collagen‐induced arthritis (CIA). We show that SJMHE1 not only modulates the cytokine production of murine macrophage (MΦ) and dendritic cell but also affects cytokine production upon coculturing with allogeneic CD4^+^ T cell. SJMHE1 potently inhibits the cytokine response to TLR ligands lipopolysaccharide (LPS), CpG oligodeoxynucleotides (CpG) or resiquimod (R848) from mouse splenocytes, and human PBMCs stimulated by LPS. Furthermore, SJMHE1 suppressed clinical signs of CIA in mice and blocked joint erosion progression. This effect was mediated by downregulation of key cytokines involved in the pathogenesis of CIA, such as interferon‐γ (IFN‐γ), tumour necrosis factor‐α (TNF‐α), interleukin (IL)‐6, IL‐17, and IL‐22 and up‐regulation of the inhibitory cytokine IL‐10, *Tgf‐*β*1* mRNA, and CD4^+^ CD25^+^Foxp3^+^ Tregs. This study provides new evidence that the peptide from *S. japonicum*, which is the ‘safe’ selective generation of small molecule peptide that has evolved during host–parasite interactions, is of great value in the search for novel anti‐inflammatory agents and therapeutic targets for autoimmune diseases.

## Introduction

Schistosomiasis is an important tropical disease that affects approximately 200 million people worldwide. Schistosomes have evolved multiple immunomodulatory mechanisms to evade host immune responses to ensure their survival without eliciting lethal immunopathology 1, 2. Infection with schistosomes or exposure to schistosome‐derived antigens prevents a range of autoimmune disorders and allergy in experimental animal models 3, including type 1 diabetes in non‐obese diabetic mice 3, 4 and experimental allergic encephalomyelitis 5, 6. The suppression mechanism is associated with immunomodulation induced by the interactions of schistosome‐derived antigens with immune cells of hosts 2. Some phospholipids or glycoproteins from helminthes ligate toll‐like receptors (TLRs), thereby inducing an anti‐inflammatory phenotype 7, 8. A phosphorylcholine‐containing glycoprotein called ES‐62, which comes from the nematode *Acanthocheilonema viteae*, modulates antigen‐presenting cell activation by a variety of TLR ligands 9. A lipid fraction from *Schistosoma mansoni* eggs that contain lysophosphatidylserine induces the activation of dendritic cells (DCs) that promote Th2 and regulatory T‐cell development in a TLR2‐dependent mechanism 10. Our previous work showed that an HSP60‐derived peptide called SJMHE1 from *S. japonicum* could induce the production of CD4^+^CD25^+^ Tregs *in vivo* and *in vitro*. Adoptively transferred SJMHE1‐induced CD4^+^CD25^+^ T cells inhibited delayed‐type hypersensitivity (DTH) in mice 11. Furthermore, SJMHE1 suppressed DTH responses in mice by co‐immunization with ovalbumin (OVA) at the time of priming. This suppression was mediated by CD4^+^CD25^+^ Tregs, IL‐10, and TGF‐β1 12, thereby implying a broad immunosuppressive action.

In this study, we assessed the ability of SJMHE1 to manipulate cytokine production in murine and human immune cells by exposing them to LPS, CpG, or R848. Data show that SJMHE1 not only induces murine MΦs/DCs and MΦs/DCs‐mixed allogeneic CD4^+^ T cell to produce anti‐inflammatory cytokines and suppress mouse splenocytes to produce pro‐inflammatory cytokine exposed by LPS, CpG, or R848; SJMHE1 also inhibits human PBMC to release pro‐inflammatory cytokines *via* LPS stimulation. Furthermore, mice treated with SJMHE1 were protected against arthritis induced by bovine II‐type collagen. As a small molecule peptide, SJMHE1 exerts immunosuppressive and anti‐inflammatory effects. Moreover, it is a potential novel drug candidate for ameliorating immunopathology.

## Materials and methods

### Ethics statement

Animal experiments were performed in strict accordance with the Regulations for the Administration of Affairs Concerning Experimental Animals (1988.11.1). All efforts were made to minimize the suffering of the animals. All animal procedures were approved by the Institutional Animal Care and Use Committee (IACUC) of Jiangsu University for the use of laboratory animals (Permit Number: JSU 14‐10).

### Mice and cell line

Six‐week‐old C57BL/6 female mice were provided by the Center of Experimental Animals (Nanjing University, Nanjing, China). Six‐week‐old male DBA/1J mice were purchased from the SLAC Laboratory (Shanghai, China) and bred under specific pathogen‐free conditions in the Animal Care Facility of the University of Jiangsu. The experimental protocol was approved by the Institutional Animal Care and Use Committee (IACUC) as previously described 13, 14.

The mouse macrophage cell line RAW264.7 was purchased from the American Type Culture Collection (Manassas, VA, USA).

### Peptides and TLR ligands

SjHSP60 437‐460 (SJMHE1) (VPGGGTALLRCIPVLDTLSTKNED) was synthesized and purified by Top‐peptide (Shanghai, China) and was pre‐treated with polymyxin B‐agarose to exclude possible LPS contamination as described previously 15. The purity of the peptides was greater than 99%, as determined by mass spectrometry.

The TLR9 ligand CpG oligodeoxynucleotides (ODN) 1826 (CpG; 5′‐TCCATGACGTTCCTGACGTT‐3′), which has a nuclease‐resistant phosphorothioate backbone and undetectable endotoxin, was purchased from Coley Pharmaceutical Group (Wellesley, MA, USA). The TLR7/8 ligand R848 was obtained from Invivogen (Toulouse, France). The TLR4 ligand LPS was obtained from Sigma‐Aldrich (St. Louis, MO, USA).

### Cell isolation

Splenocytes were prepared by tearing apart spleens from mice in PBS that contained 1% FCS and 1% ethylenediaminetetraacetic acid followed by red blood cell lysis with Tris ammonium chloride buffer. CD4^+^ T cells were purified from splenocytes with a CD4^+^ T cell negative‐isolation kit (Miltenyi Biotec, Auburn, CA, USA) and a magnetic activated cell sorter according to the manufacturer's recommendations (>97% CD4^+^ T cells by flow cytometric analysis). Murine bone marrow‐derived DCs (BMDCs) were generated from C57BL/6 mice as previously described 11.

Human peripheral blood mononuclear cells (PBMCs) were isolated from five healthy volunteers through density gradient centrifugation using Ficoll‐Hypaque (Amersham Pharmacia Biotech, Uppsala, Sweden) as previously described 16.

### Cell culture

For *in vitro* antigen stimulation assays, 2 × 10^5^ BMDCs/well were cultured in 24‐well plates in triplicate and pulsed with 0.1 μg/ml SJMHE1 (DC_SJMHE1_), in medium alone (DC_medium_) for 8 days, or in the presence of 1 μg/ml LPS from *Escherichia coli* 055:B5 (Sigma‐Aldrich) (DC_LPS_) for the last 48 hrs of an 8‐day culture. In addition, 2 × 10^5^ RAW264.7 cells/well were pulsed with either 0.1 μg/ml SJMHE1 (MΦ_SJMHE1_), 1 μg/ml *E. coli* LPS (MΦ_LPS_), or medium alone (MΦ_medium_) for 24 hrs. The supernatants were collected for cytokine detection.

To observe the effect of CD4^+^ T cells *in vitro*, 2 × 10^5^ allogeneic CD4^+^ T cells/well were purified from naive mice and cultured with or without 5 × 10^4^ MΦ_medium_/DC_medium_ cells, MΦ_SJMHE1_/DC_SJMHE1_ cells or MΦ_LPS_/DC_LPS_ cells, respectively. The supernatants were collected 48 hrs after coculturing to detect cytokines. Cytokine contents in the supernatants of MΦ/DC cultures or MΦ/DC‐CD4^+^ T‐cell cocultures were determined by ELISA (Bender Med Systems, Vienna, Austria) in accordance with the manufacturer's protocol.

For TLR ligand stimulation assays, 2 × 10^5^ splenocytes per well from naive mice were stimulated with 1 μg/ml LPS, 3 μg/ml CpG, 3 μg/ml R848, or in the presence of 1 μg/ml SJMHE1 for 48 hrs. The supernatants were collected to measure cytokines. Peripheral blood mononuclear cells from healthy volunteers were stimulated with 1 μg/ml LPS or in the presence of 1 μg/ml SJMHE1. Cultures were incubated at 37°C for 2 days and the supernatants were collected to detect cytokines. Cytokine production in the supernatants of splenocytes or PBMCs stimulated by TLR ligands was detected using the FlowCytomix Mouse or Human Cytokine Kit (Bender Med Systems) according to the manufacturer's instructions.

### Induction of and assessment of collagen type II‐induced arthritis

Collagen‐induced arthritis (CIA) was induced in 6‐ to 8‐week‐old male DBA/1 mice through 200 μg intradermal injections of bovine type II collagen (CII) (Chondrex, Redmond, WA, USA) in accordance with a previous adaptation of standard protocol 17. Each mouse received 100 μl injections that contained 200 μg of CII and 200 μg of inactivated *Mycobacterium tuberculosis* (H37Ra; Difco, Detroit, MI, USA) in IFA on days 0 and 21. Mice were treated on days −7, 7, and 14 with PBS, 10 μg SJMHE1, or 10 μg OVA_323‐339_. In addition, six naive DBA/1J mice were used as controls and killed on day 44.

The clinical disease activity of the CIA was assessed every other day between days 21 and 44 by two‐blinded observers with the use of a three‐point scale for each paw: 0 = no change; 1 = mild swelling and/or erythema; 2 = moderate swelling and erythema; and 3 = marked swelling and erythema. The total score for clinical disease activity was based on all four paws, with a maximum score of 12 for each animal 17. Right hindlimbs were surgically removed from all mice on day 44 and fixed in 10% buffered formalin; the tissue samples were prepared and histological analyses were performed as previously described 18. The tissue sections were scored by Pathology laboratory personnel who were blinded to the treatment protocol. The scoring system utilized was as previously described 17.

### Cytokine determination

Spleens were removed from mice on day 44, and 2 × 10^5^ splenocytes per well were cultured for 48 hrs at 37°C in the presence of 50 μg/ml bovine CII. The supernatants were then collected and observed for cytokines *via* FlowCytomix Mouse Cytokine Kit (Bender Med Systems) according to the manufacturer's instructions.

### Antibody detection in the sera of mice with CIA

Serum was obtained from mice on day 44, and standard ELISAs were performed using CII as the antigen source for antibody detection as previously described 17. The ELISA plates were coated with 5 μg/ml of CII and kept at 4°C overnight. Plates were washed and developed using tetramethylbenzidine substrate (Sigma‐Aldrich). To analyse IgG, IgG1 and IgG2a, mouse‐specific secondary antibodies (Bio‐Rad Laboratories Inc, Hercules, CA, USA) were used at a dilution of 1:1000. The enzymatic reaction was stopped with 1 N H_2_SO_4_ and plates were read at a 450 nm wavelength.

### RNA extraction and quantitative PCR

Spleen cells were obtained from mice on day 44, and total RNA was extracted using Trizol reagent (Invitrogen, Carlsbad, CA, USA), according to the manufacturer's protocols. For measurement of *Tgf‐*β*1* mRNA, 500 ng of total RNA from spleen cells of mice were reverse‐transcribed using the All‐in‐one^™^ First‐Strand cDNA Synthesis kit (Genecopoeia, Germantown, MD, USA) according to the manufacturer's manual. All‐in‐one^™^ qPCR Primer sets for *Tgf‐*β*1* (cat. no. MQP030343) were used, and mouse β‐actin (cat. no. MQP026493) was used as an endogenous control for sample normalization. Real‐time PCR was performed with All‐in‐one^™^ qPCR Mix (Genecopoeia) in a CFX96^™^ Real‐Time system (Bio‐Rad Laboratories Inc). All reactions were done and normalized to the expression of β‐actin based on the previous description 16.

### Statistical analysis

Statistical analyses were performed with GraphPad Prism 5.01 (GraphPad Software, 2007, La Jolla, CA, USA). Results were expressed as mean ± S.E.M. Statistical comparisons between groups were conducted using one‐way anova followed by Bonferroni test. Histopathology scores were analysed by one‐way anova. *P* < 0.05 was considered statistically significant.

## Results

### SJMHE1 induces mouse MΦs/BMDCs to produce anti‐inflammatory cytokines *in vitro*

**Figure 1:**
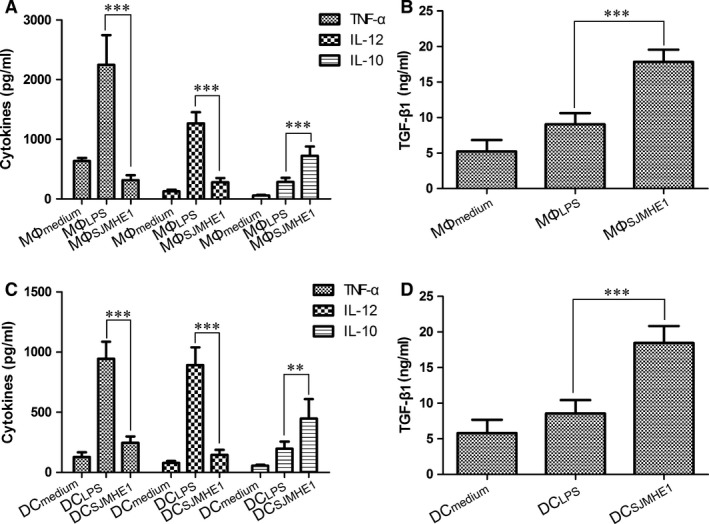
SJMHE1 induces mouse MΦs/BMDCs to produce anti‐inflammatory cytokines *in vitro*. MΦ (RAW264.7 cells) or BMDC were treated with SJMHE1 (MΦ_SJMHE__1_, BMDC_SJMHE__1_), LPS (MΦ_LPS__,_ BMDC_LPS_), or medium (MΦ_medium_, BMDC_medium_) as described in Materials and Methods. (**A** and **B**) The cytokines in culture supernatants of MΦ or DC (**C** and **D**) were analysed *via* ELISA. Each result is the mean ± S.E.M. of three experiments performed in triplicate wells. Significance analysed by one‐way anova with Bonferroni test. **P < 0.01; ***P < 0.001.

To determine the effects of SJMHE1 on cytokine production from mouse MΦs and BMDCs, we treated MΦs/BMDCs with medium, LPS, or SJMHE1, and observed the cytokines in the supernatant *via* ELISA. Consistent with our previous results 11, MΦs or BMDCs produced high levels of inflammatory cytokines with LPS stimulation, as shown in Figure 1. However, SJMHE1 treatment resulted in lower amounts of pro‐inflammatory cytokine (TNF‐α and IL‐12) but released significant levels of anti‐inflammatory cytokines IL‐10 and TGF‐β1. These results indicate that SJMHE1, in contrast with LPS, does not induce pro‐inflammatory cytokine release but induces significant levels of anti‐inflammatory cytokines following *in vitro* treatment of macrophages and DC.

### SJMHE1‐treated MΦs/BMDCs cocultured with allogeneic CD4^+^ T cell induces anti‐inflammatory cytokine production *in vitro*

**Figure 2:**
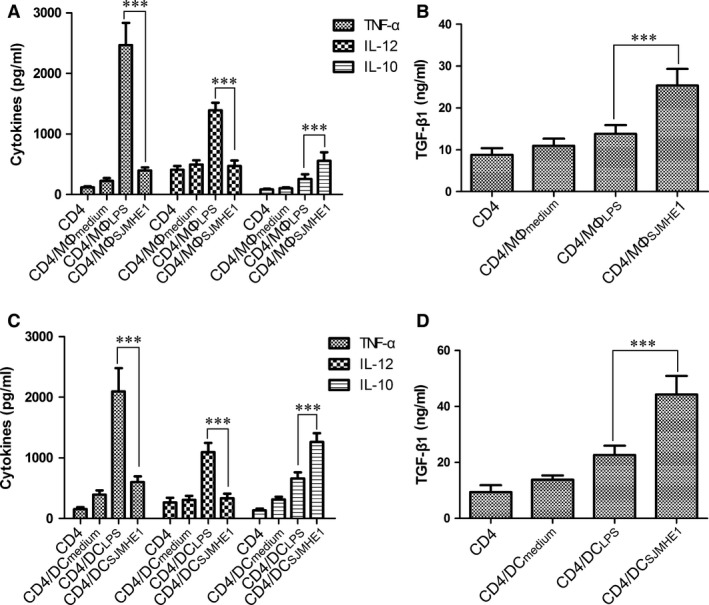
SJMHE1‐treated MΦs/BMDCs cocultured with allogeneic CD4^+^ T cell induces anti‐inflammatory cytokine production *in vitro*. MΦ (RAW264.7 cells) or BMDC were treated with SJMHE1 (MΦ_SJMHE__1_, BMDC_SJMHE__1_), LPS (MΦ_LPS_, BMDC_LPS_), or medium (MΦ_medium,_ BMDC_medium_) as described in Materials and Methods. CD4^+^ T cells were purified from naive C57BL/6 mice and cultured with the MΦ or DC. After 3 days of coculture, (**A** and **B**) The cytokines in culture supernatants of coculture with MΦ or DC (**C** and **D**) were analysed *via* ELISA. Each result is the mean ± S.E.M. of three experiments performed in triplicate wells. Significance analysed by one‐way anova with Bonferroni test. ***P < 0.001.

Our previous reports indicated that the capacity of SJMHE1‐treated MΦs or BMDCs to prime allogeneic CD4^+^ T cell for proliferation was weaker compared with OVA^323‐329^ or LPS‐treated MΦs or BMDCs 11. To determine whether the cytokine production was different and affected CD4^+^ T‐cell proliferation, we observed the cytokines in the supernatant of cocultured SJMHE1‐treated MΦs or BMDCs with alloreactive CD4^+^ T cells. As shown in Figure 2, the coculture of LPS‐treated MΦs or BMDCs and CD4^+^ T cells produced high levels of TNF‐α and IL‐12 (Fig. 2A and C). By contrast, SJMHE1‐treated MΦs or BMDCs cocultured with CD4^+^ T cells significantly reduced TNF‐α and IL‐12 productions and simultaneously increased IL‐10 and TGF‐β1 releases (Fig. 2). Thus, high levels of IL‐10 and TGF‐β1 may reduce CD4^+^ T‐cell proliferation when cocultured with SJMHE1‐treated MΦs or BMDCs.

### SJMHE1 inhibits cytokine production of mouse splenocytes in response to TLR ligands

**Figure 3:**
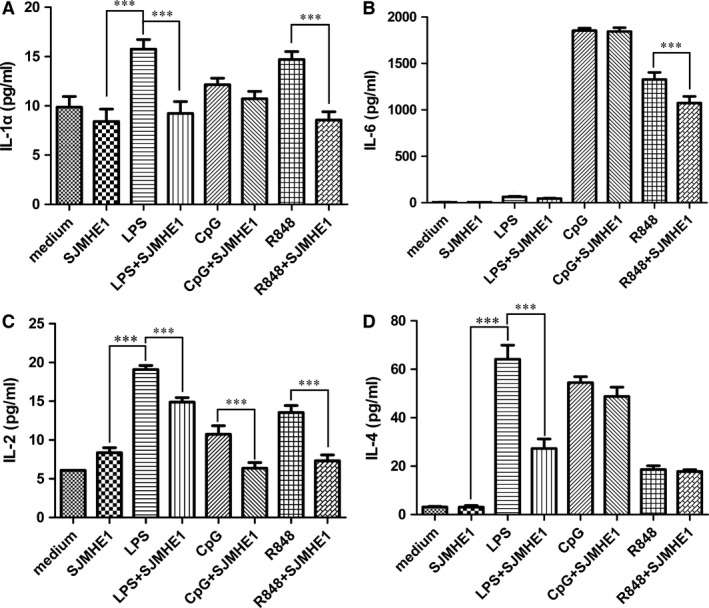
SJMHE1 inhibits cytokine production of mouse splenocytes in response to TLR ligands. Splenocytes from naive C57BL/6 mice were stimulated with 1 μg/ml LPS, 3 μg/ml CpG, and 3 μg/ml R848 either alone or in the presence of 1 μg/ml SJMHE1. Supernatants were collected after 2 days and tested for IL‐1α (**A**), IL‐6 (**B**), IL‐2 (**C**), and IL‐4 (**D**). Each result is the mean ± S.E.M. of two experiments performed in triplicate wells. Significance analysed by one‐way anova with Bonferroni test. ***P < 0.001.

Worm molecules could modulate TLR signalling to induce an anti‐inflammatory response 19. Previous work has demonstrated that SEA can suppress TLR ligand‐induced DC activation by modulating pro‐inflammatory cytokine production 20, 21. SJMHE1 is a peptide from SjHSP60, which is a component of SEA. To determine whether SJMHE1 would suppress TLR ligand‐induced cytokine production, splenocytes were isolated from naive C57BL/6 and stimulated with LPS (TLR4 ligand), the TLR9 ligand CpG, the TLR7/8 ligand R848 alone, or in the presence of SJMHE1. As shown in Figure 3, LPS, CpG or R848 alone induced high levels of IL‐1α, IL‐2, IL‐6 and IL‐4. By contrast, SJMHE1 limited cytokine production. However, when splenocytes were pulsed simultaneously with SJMHE1 and LPS, CpG, or R848, the up‐regulation of cytokines was significantly inhibited. SJMHE1 suppressed IL‐1α, and IL‐2 production induced by either LPS or R848; and SJMHE1 inhibited IL‐2 production induced by LPS, CpG, or R848. Furthermore, SJMHE1 can suppress R848‐induced IL‐6 release and inhibit LPS‐induced IL‐4 production. These results demonstrate that SJMHE1 suppresses the cytokine production of mouse splenocytes stimulated by TLR ligands but with differences in apparent efficiency.

### SJMHE1 also inhibits the cytokine production from human PBMCs with LPS stimulation

**Figure 4:**
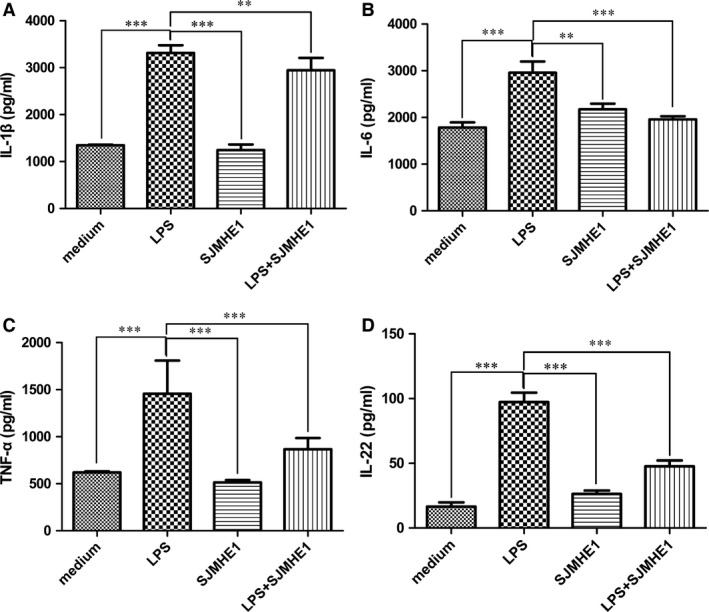
SJMHE1 inhibits the cytokine production from human peripheral blood mononuclear cells with LPS stimulation. Human PBMC from healthy volunteers were stimulated with 1 μg/ml LPS or in the presence of 1 μg/ml SJMHE1. Supernatants were collected after 2 days and tested for IL‐1β (**A**), IL‐6 (**B**), TNF‐α (**C**) and IL‐22 (**D**). Each result is the mean ± S.E.M. of two experiments performed in triplicate wells. Significance analysed by one‐way anova with Bonferroni test. **P < 0.01; ***P < 0.001.

To investigate the effects of SJMHE1 on cytokine production of human cells, we focused on LPS as a stimulus, given that triggering TLR4 *via* LPS is one of the most well‐characterized pathways that lead to cytokine release 22. We analysed the cytokine production of human PBMCs stimulated with LPS or in the presence of SJMHE1. As shown in Figure 4, LPS induced a higher level of pro‐inflammatory cytokines compared with the medium. By contrast, SJMHE1 alone almost did not stimulate cytokine production. However, when PBMCs were pulsed simultaneously with SJMHE1 and LPS, SJMHE1 potently suppressed LPS‐induced productions of IL‐1β, IL‐6, TNF‐α and IL‐22. These results suggest that SJMHE1 also inhibits human PBMCs to produce inflammatory cytokines and strongly imply that SJMHE1 may be a possible therapeutic molecule for autoimmune disease treatments.

### SJMHE1 suppresses CIA in mouse models

**Figure 5:**
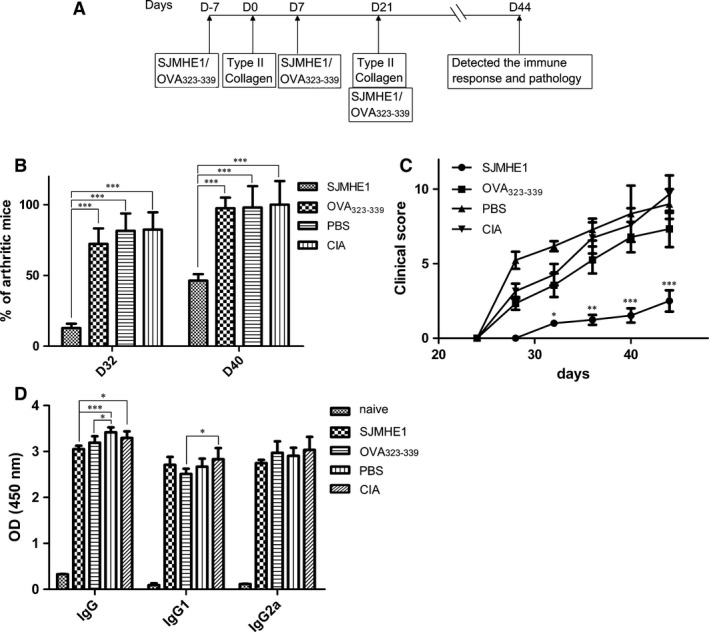
SJMHE1 treatment reduces the severity and incidence of CIA mice. DBA/1J mice (6 mice per group) were injected with 200 μg of CII and 200 μg of inactivated *Mycobacterium tuberculosis* in IFA on days 0 and 21, and mice were treated on days −7, 7, and 14 with PBS, 10 μg SJMHE1 and 10 μg OVA_323‐339_. The mice were killed on day 44. (**A**) Schedule of SJMHE1 treatment. (**B**) Percentage of arthritic mice on days 32 and 40 after primary immunization. (**C**) Clinical score for each group at each time‐point. Bars represent the mean ± S.E.M. (*n* = 6 per group) of 18 mice from three‐independent experiments. (**D**) IgG, IgG1 and IgG2a responses in mice. Antibody responses to CII (5 μg/ml) were determined *via* ELISA. Data are expressed as the mean ± S.E.M. (*n* = 6 per group) of 12 mice from two‐independent experiments. Significance analysed by one‐way anova with Bonferroni test. *P < 0.05; **P < 0.01; ***P < 0.001.

SJMHE1 showed a broad spectrum of inhibitory effects including the suppression of many pro‐inflammatory cytokines from mouse splenocytes and human PBMCs stimulated by TLR ligands. These inhibitory activities may suggest the effectiveness of SJMHE1 against inflammatory and immune disorders. To examine the effects of SJMHE1 in CIA progression, DBA/1 mice were treated with bovine CII (days 0 and 21). The treatment regimen is illustrated in Figure 5A. As shown in Figure 5B, CIA developed rapidly in DBA/1 mice immunized with CII plus adjuvant, and clinical signs, *e.g*. periarticular erythema and oedema, first appeared in the hind paws 24–28 days after primary immunization with 100% incidence at day 40. Paw erythema and swelling increased in frequency and severity in a time‐dependent manner (Fig. 5C). However, SJMHE1 treatment resulted in significantly less joint inflammation, shown by a significantly lower incidence of arthritis (*P* < 0.001) and lower clinical scores over time than those in the OVA_323‐339_, PBS and CIA control groups.

Given that a strong B‐cell response is activated in CIA, resulting in anti‐CII IgG production, which is involved in the pathogenesis and promotion of arthritis development 23. Antibody responses to CII in CIA mice treated with SJMHE1, OVA_323‐339_ or PBS were measured in sera obtained on day 44. As shown in Figure 5D, apart from naive mice, IgG, IgG1 and IgG2a responses were observed in CII‐immunized mice, regardless of SJMHE1, OVA_323‐339_ or PBS treatments. However, SJMHE1 treatment produced a more significant suppression of anti‐CII IgG production compared with PBS‐treated (*P* < 0.001) or CIA mice (*P* < 0.05; Fig. 5D).

**Figure 6:**
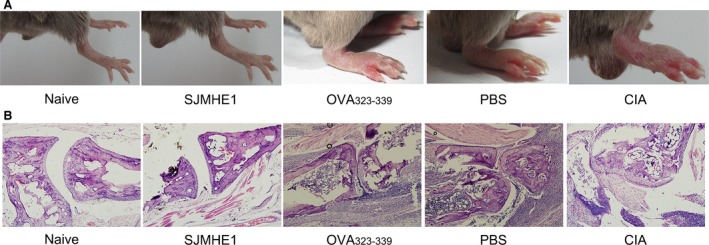
SJMHE1 treatment reduces the clinical signs and joint histology of CIA mice. On day 44, the mice were killed and their joints were analysed for histology. (**A**) Arthritic hind paws for each group are shown. (**B**) The histology (haematoxylin–eosin staining) for each group is shown.

Furthermore, SJMHE1 treatment reduced arthritis severity, as indicated by paw thickness and redness assessments through macroscopic observation of the hind paws on day 44, as shown in Figure 6A; the difference in the image colour is not significant and is simply because of lighting differences. Moreover, SJMHE1 reduced the histopathological alterations at the joint level. As shown in Figure 6B, the joints of naive mice appeared histologically normal with no significant inflammatory cell infiltration or cartilage‐bone destruction. By contrast, OVA_323‐339_, PBS‐treated or CIA mice showed massive inflammatory cell infiltration and cartilage‐bone destruction. However, SJMHE1‐treated mice exhibited minimal cellular infiltration and cartilage‐bone damage. Table 1 shows the histopathological scoring of the paws from the five groups. Mice that received SJMHE1 peptide injection had significantly reduced inflammation, pannus, and cartilage and bone damage scores in histopathological examination of paws. These results indicate that SJMHE1 can protect mice from severe autoimmune‐mediated inflammation and bone destruction.

**Table 1:** Histopathology scores in mice with collagen‐induced arthritis

Parameter	Treatment				
	SJMHE1	OVA_323‐339_	PBS	CIA	P
Inflammation	0.4 ± 0.2	1.8 ± 0.4	2.1 ± 0.5	2.2 ± 0.4	<0.01
Pannus	0.3 ± 0.2	1.2 ± 0.4	1.5 ± 0.3	1.6 ± 0.4	<0.05
Cartilage damage	0.4 ± 0.2	1.5 ± 0.4	1.7 ± 0.5	2.0 ± 0.4	<0.05
Bone damage	0.2 ± 0.1	0.9 ± 0.3	1.0 ± 0.2	1.1 ± 0.3	<0.05
Total score	1.3 ± 0.7	5.5 ± 0.6	6.2 ± 0.5	6.8 ± 0.9	<0.0001

Results are expressed as means ± S.E.M. The mean values were calculated using results from 12 mice from two‐independent experiments (*n* = 6). Significance analysed by one‐way anova.

### SJMHE1 modulates the production of cytokines in mouse splenocytes from CIA mice

**Figure 7:**
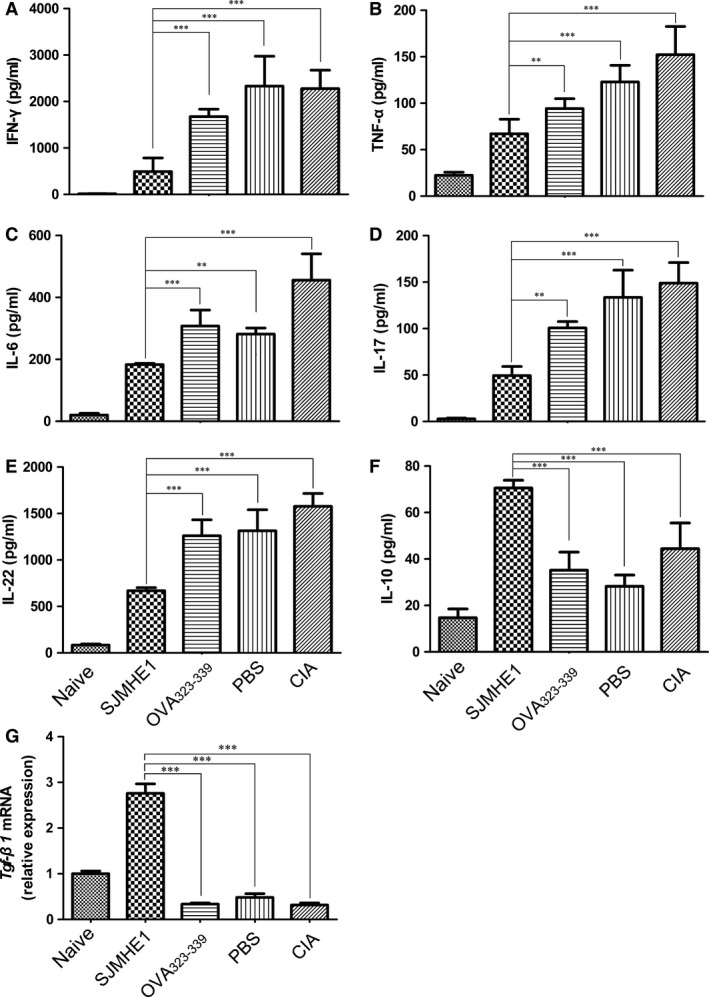
SJMHE1 modulates the production of cytokines in mouse splenocytes from CIA mice. On day 44, the mice were killed, and the splenocytes from each mouse were incubated in the presence of CII (50 μg/ml) or control media for 2 days at a volume of 200 μl in 96‐well plates. Supernatants were collected after 48 hrs and tested for IFN‐γ (**A**), TNF‐α (**B**), IL‐6 (**C**), IL‐17 (**D**), IL‐22 (**E**) and IL‐10 (**F**). Bars represent the mean ± S.E.M. (*n* = 6 per group) of 18 mice from three‐independent experiments performed in triplicate wells. Cell pellets were collected after 48 hrs and mRNA expression of *Tgf‐*β*1* was detected (**G**). The expression of *Tgf‐*β*1* mRNA in mice with different treatment as relative levels compared with naive controls (*n* = 6). Data are expressed as the mean ± S.E.M. Significance analysed by one‐way anova with Bonferroni test. **P < 0.01; ***P < 0.01.

Pro‐inflammatory cytokines, such as IL‐1β, TNF‐α and IL‐6, are crucial in the pathogenesis of arthritis during inflammatory responses 24, 25, 26. Many reports showed the prominent role of Th cells in producing IL‐17 in the pathogenesis of human rheumatoid arthritis (RA); moreover, IL‐17 levels were elevated in the synovium of RA patients during pathogenesis of RA 27, 28. SJMHE1 suppressed the progression of CIA, a mouse model of RA. Therefore, we evaluated the secretion of pro‐inflammatory cytokines *via* CII‐stimulated splenocytes *ex vivo*. Figure 7 shows that *ex vivo* restimulation with CII increased IFN‐γ, TNF‐α, IL‐6, IL‐17 and IL‐22 in OVA_323‐339‐_, PBS‐treated, and CIA mice. However, the production of these cytokines in the supernatant of SJMHE1‐treated mice splenocytes was significantly reduced compared with those in mice treated with OVA_323‐339‐_, PBS‐treated, or CIA alone. By contrast, the levels of anti‐inflammatory cytokine IL‐10 and *Tgf‐*β*1* mRNA were significantly increased through SJMHE1 treatment in CIA mice. These results suggest that SJMHE1 modulates CIA by inhibiting the production of pro‐inflammatory cytokines.

### SJMHE1 treatment increases the population of CD4^+^CD25^+^Foxp3^+^ Tregs in CIA mice

**Figure 8:**
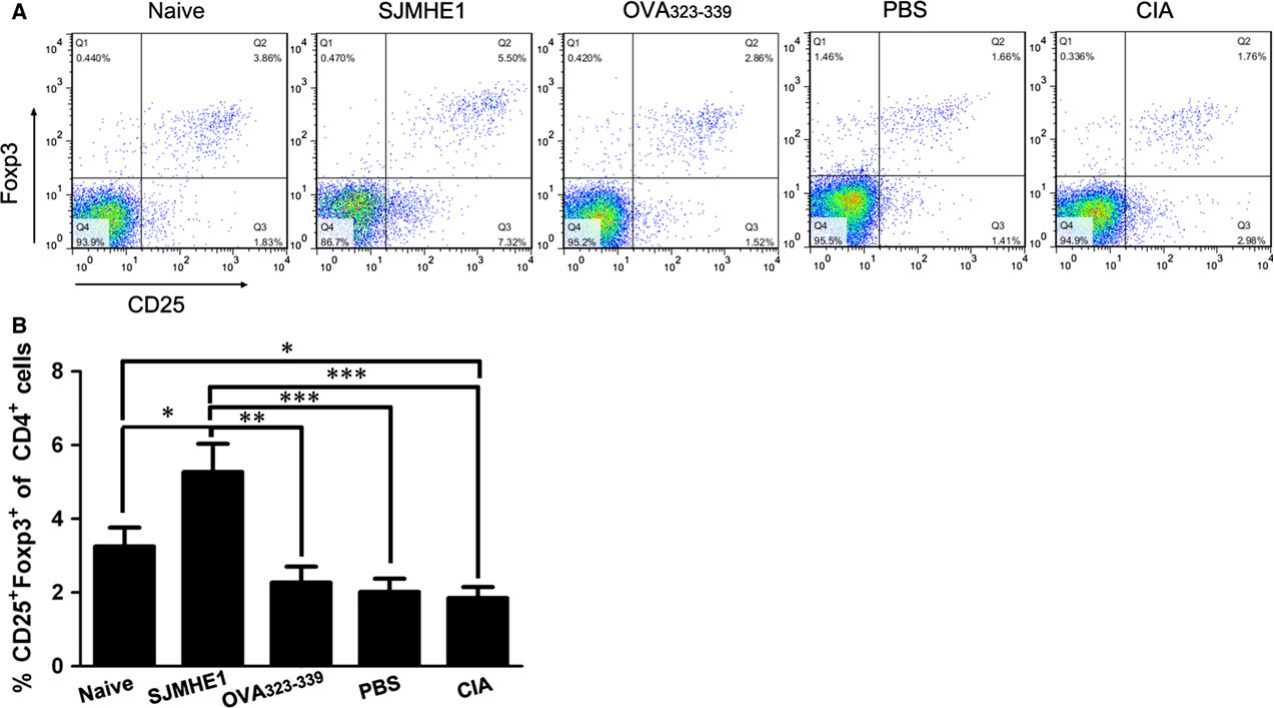
SJMHE1 treatment increases the population of CD4^+^ CD25^+^Foxp3^+^ Tregs in CIA mice. On day 44, the mice were killed, and the splenocytes from each mouse were analysed by flow cytometry for CD3, CD4, CD25, and Foxp3. (**A**) Data are representative of the experiments; (**B**) The percentage of CD4^+^ CD25^+^Foxp3^+^ T cells. Data are expressed as the mean ± S.E.M. of 12 mice from two‐independent experiments. Significance analysed by one‐way anova with Bonferroni test. *P < 0.05; **P < 0.01; ***P < 0.01.

Defects in CD4^+^CD25^+^ Tregs have been observed in RA patients 16, and Treg plays a pivotal role in the regulation of auto‐reactive T‐cell activation in CIA 29. Our previous study demonstrated that SJMHE1 treatment increases CD4^+^CD25^+^ Tregs *in vivo* and *in vitro* 11, as well as generates CD4^+^CD25^+^ Tregs to suppress DTH responses 12. We examined whether SJMHE1 inhibits arthritis and modulates cytokine production in CIA mice by increasing the number of Tregs during treatment. We then tested the CD4^+^CD25^+^Foxp3^+^ Tregs from CIA mice treated with PBS, SJMHE1, and OVA_323‐339_. As shown in Figure 8, compared with the naive mice or the PBS‐ and OVA_323‐339_‐injected mice, SJMHE1 treatment increased the proportion of CD4^+^CD25^+^Foxp3^+^ T cells significantly. Consistent with results described previously 30, the frequencies of CD4^+^CD25^+^ Tregs were decreased in CIA mice compared with naive mice. These results suggest that SJMHE1 generated CD4^+^CD25^+^ Foxp3^+^ Tregs, which might mediate protection against CIA.

## Discussion

Increasing evidence shows that infection with microorganisms, particularly helminthes, can protect against allergic and autoimmune inflammatory disorders, as suggested by the hygiene hypothesis 31. As a result, the potential for exploiting worm‐based immunomodulation to treat inflammatory conditions has received increasing interest 19. However, most helminth antigens reportedly contain a large mixture of proteins, glycol‐proteins, and glycol‐lipids or whole protein 7, 8, 32. These mixture molecules or whole proteins may cross‐link adjacent IgE on mast cells and basophils, or activate pathogenic B and T cells, thereby exacerbating the allergy or autoimmune response 33, 34. By contrast, a short linear peptide generally avoids the above deficiencies and can antagonize the inflammatory responses of allergy and autoimmune diseases 33, 34. In this study, we demonstrated that SJMHE1, which is a HSP60‐derived peptide from *S. japonicum*, suppressed the pro‐inflammatory cytokine production from mouse and human immune cells stimulated by TLR ligands, as well as ameliorated CIA in mice.

Helminth‐derived molecules trigger innate cells first and then initiate the subsequent adaptive response. In this regard, extensive research has focused on the responses of classical innate cells, such as macrophages (MΦ) and DC. Consistent with our previous findings and the failure of other helminth antigens to induce conventional pro‐inflammatory responses 11, 21, 35, SJMHE1‐treated MΦ and DC reduce the production of TNF‐α and IL‐12, and increase the release of IL‐10 and TGF‐β1. Furthermore, SJMHE1‐treated MΦ and DC can modulate the CD4^+^ T cell responses and dampen pro‐inflammatory cytokine release while increasing the anti‐inflammatory cytokine production upon coculturing with allogeneic CD4^+^ T cell. These results may explain why helminth infection results in impaired Th1 development and drives Th2 or regulatory responses 36, 37.

A wealth of evidence from both *in vitro* and murine models indicates that helminth parasites or their secreted products can modulate and suppress DC function 21, 38. Monocyte‐derived DC (mDC) isolated from Schistosoma‐infected subjects has a reduced capacity to respond to TLR ligands and to initiate T helper cell responses, and it reduces IL‐12 and IL‐6 production in response to TLR ligands LPS and R848 39. Consistent with these results, SJMHE1 can suppress the production of IL‐1α, IL‐6, IL‐2 and IL‐4 from mouse splenocytes stimulated by LPS, CpG or R848, although selective inhibition to up‐regulate pro‐inflammatory cytokines was observed after stimulation with LPS, CpG, and R848. This finding suggests that the suppress response may not be TLR‐specific but is rather a more general phenomenon. Furthermore, SJMHE1 can modulate the immune system to suppress host‐protective (and possibly pathological) pro‐inflammatory responses; this ability is similar to that of other helminth‐derived immunomodulators 32.

Apart from inhibiting the pro‐inflammatory cytokines from mice splenocytes stimulated by TLR ligands, SJMHE1 also effectively inhibited the secretion of pro‐inflammatory cytokines from human PBMCs stimulated by LPS. The immunomodulatory effect of SJMHE1 in the humans is similar in mice. These results are consistent with another Schistosoma antigens Sm16, which can potently inhibit the LPS response to human blood and monocytic cells 35. However, mouse splenocytes and human PBMCs were mixture and the suppression effect of cytokines suggested SJMHE1 had a broad immunosuppressive action, which can inhibit various T cell and antigen presenting cell‐associated cytokines induced by TLR ligands, including Th1 cytokines (IL‐2 and TNF‐α), Th2 cytokines (IL‐4 and IL‐6), and macrophage derived cytokines (TNF‐α and IL‐1β).

The inhibitory effect of SJMHE1 on pro‐inflammatory cytokines suggests that it may affect the outcome of inflammatory diseases in murine model systems. In this study, we tested the effects of SJMHE1 treatment on CIA progression in mice. CIA is one of the most widely used animal models for studying RA and testing potential therapeutic agents because of its pathologic, immunological and clinical similarities to human RA 40. SJMHE1 improved clinical symptoms and decreased the incidence and severity of CIA in mice. Anti‐collagen antibodies reflecting the arthritis development are involved in the inflammatory attack against joints 23. Consistent with the reduction in clinical signs of disease, SJMHE1‐induced reduction in IgG production confirmed and supported the beneficial role of SJMHE1 in CIA mice. Histopathological analysis of joints showed that cartilage pathology and bone destruction were reduced in SJMHE1‐treated animals.

The pathogenesis of RA is complex and largely unknown. Th1, Th17, IFN‐γ, TNF‐α, IL‐1β, IL‐6, IL‐17 and various inflammatory cytokines are important in the hierarchy of RA processes 24, 41; whereas IL‐10 and TGF‐β1 have potent anti‐inflammatory effects and suppress cartilage and bone pathologies in RA 42, 43. The systemic balance of cytokines has been altered in previous studies by blocking TNF‐α and IL‐1β *via* biological agents, such as anti‐TNF‐α or IL‐1 inhibitors 44, 45. Clinical trials that tested the effects of anti‐cytokine therapy showed marked alleviation in pain, swelling and the progression of joint destruction 46, 47. However, this therapy produces a series of side effects, *e.g*. anti‐TNF therapy can reactivate latent tuberculosis 48. Interestingly, SJMHE1 treatment markedly reduced either IFN‐γ (*i.e*. cytokines produced by Th1 cells), IL‐17 and IL‐22 (*i.e*. cytokines produced by Th17 cells), or TNF‐α and IL‐6 (*i.e*. cytokines produced by activated macrophages and T cells), which were dominant in inducing inflammation and bone erosion 24, 25, 49; although the action of IL‐22 in inflammatory arthritis is controversial. Multiple studies have reported that IL‐22 has a pathogenic role in RA 50, whereas other studies showed that IL‐22 reduced the progression of arthritis in mice with CIA 51. These findings suggested that IL‐22 has pathogenic or protective actions in inflammatory arthritis, depending on the different phases of disease development 50. In this study, we observed an increase in IL‐10, *Tgf‐*β*1* mRNA, and CD4^+^CD25^+^Foxp3^+^ Tregs in the splenocytes of mice treated with SJMHE1, which likely contributed to the dampened production of pro‐inflammatory cytokines and protection against CIA. Therefore, SJMHE1 is a promising new biological agent for treating CIA mice. However, this peptide induced protection against CIA whether or not it interfered with the differentiation and function of DC and/or macrophage requires further analysis as previously described 11. Maturation and activation of DC and/or macrophages are key steps in triggering the priming of auto‐reactive peripheral T cells, which then drive the development of inflammatory responses in arthritis 52; whereas tolerogenic DC and macrophages are known to induce tolerance by Tregs in various autoimmune diseases, including CIA 53. Thus, considerable work is still needed to define the mechanisms of immune alteration and determine whether therapies for allergies or autoimmunities could be developed from this peptide.

In summary, we have shown that SJMHE1, which is a peptide from *S. japonicum*, not only induces MΦ and DC from producing anti‐inflammatory cytokines, but also modulates the cytokine production of CD4^+^ T cells upon coculturing with SJMHE1‐treated MΦ and DC. Furthermore, SJMHE1 suppresses TLR ligand‐induced pro‐inflammatory cytokines from mouse splenocytes and human PBMCs *in vitro*. The active immunosuppressive activity of the SJMHE1 may have relevant in functions *in vivo*. SJMHE1 can protect mice against CIA by modulating cytokine production and up‐regulation of CD4^+^CD25^+^Foxp3^+^ Tregs. These findings open the possibility for a novel treatment of autoimmune or inflammatory diseases; these molecules may become part of our future therapeutic armamentarium.

## Conflicts of interest

The authors declare that they have no competing interests. The funding agencies played no role in the design or implementation of the study, analysis or interpretation of the data, or the preparation and submission of the manuscript.

## Author contribution

Conceived and designed the experiments: X.F.W. Performed the experiments: X.F.W., L.L., J.W. and L.Y.D. Analysed the data: Y.S., Y.L., L.S. and C.C.X. Contributed reagents/materials/analysis tools: Y.P.Z., Y.W., D.Y.C. and C.M.M. Wrote the paper: X.F.W. All authors read and approved the final manuscript.
